# Reduced Annexin A1 Secretion by ABCA1 Causes Retinal Inflammation and Ganglion Cell Apoptosis in a Murine Glaucoma Model

**DOI:** 10.3389/fncel.2018.00347

**Published:** 2018-10-11

**Authors:** Lu Li, Lingjuan Xu, Wei Chen, Xing Li, Qian Xia, Lu Zheng, Qiming Duan, Hong Zhang, Yin Zhao

**Affiliations:** ^1^Department of Ophthalmology, Tongji Medical College, Tongji Hospital, Huazhong University of Science and Technology, Wuhan, China; ^2^Department of Ophthalmology, The First Affiliated Hospital, Shihezi University School of Medicine, Shihezi, China; ^3^Key Laboratory of Neurological Diseases, Department of Neurobiology, Ministry of Education, Tongji Medical College, Huazhong University of Science and Technology, Wuhan, China; ^4^The Institute for Brain Research, Collaborative Innovation Center for Brain Science, Huazhong University of Science and Technology, Wuhan, China; ^5^Gladstone Institutes, San Francisco, CA, United States

**Keywords:** retina, ischemia/reperfusion injury, retinal ganglion cells (RGCs), ABCA1, annexin A1

## Abstract

Variants near the ATP-binding cassette transporter A1 (*ABCA1*) gene are associated with elevated intraocular pressure and newly discovered risk factors for glaucoma. Previous studies have shown an association between ABCA1 deficiency and retinal inflammation. Using a mouse model of ischemia-reperfusion (IR) induced by acute intraocular pressure elevation, we found that the retinal expression of ABCA1 protein was decreased. An induction of ABCA1 expression by liver X receptor agonist TO901317 reduced retinal ganglion cell (RGC) apoptosis after IR and promoted membrane translocation and secretion of the anti-inflammatory factor annexin A1 (ANXA1). Moreover, ABCA1 and ANXA1 co-localized in cell membranes, and the interaction domain is amino acid 196 to 274 of ANXA1 fragment. TO901317 also reduced microglia migration and activation and decreased the expression of pro-inflammatory cytokines interleukin (IL)-17A and IL-1β, which could be reversed by the ANXA1 receptor blocker Boc2. Overexpression of TANK-binding kinase 1 (*TBK1*) increased ABCA1 degradation, which was reversed by the proteasome inhibitor carbobenzoxy-L-leucyl-L-leucyl-L-leucinal (MG132). Silencing *Tbk1* with siRNA increased ABCA1 expression and promoted ANXA1 membrane translocation. These results indicate a novel IR mechanism, that leads via TBK1 activation to ABCA1 ubiquitination. This degradation decreases ANXA1 secretion, thus facilitating retinal inflammation and RGC apoptosis. Our findings suggest a potential treatment strategy to prevent RGC apoptosis in retinal ischemia and glaucoma.

## Introduction

Glaucoma is one of the leading causes of irreversible blindness worldwide, affecting nearly 60 million people (Quigley and Broman, 2006). As a neurodegenerative disorder caused by protein misfolding, glaucoma is characterized by retinal ganglion cell (RGC) death and axonal damage of the optic nerve (Levin et al., 2017; Nuzzi and Tridico, 2017). The degeneration of RGCs may be triggered by increased intraocular pressure (IOP), gene defects, high glucose levels, oxidative stress, and aging. IOP provides the best treatment target for glaucoma; however, in some patients, the RGC degeneration is progressive despite a reduction in IOP (Zhang et al., 2012).

Treatment of RGC degeneration in glaucoma is complex and still at a preclinical stage. Ding et al. created a coculture of murine induced pluripotent stem cells (iPSCs) and primary human trabecular meshwork (TM) to produce TM-like cells, which helped in controlling IOP (Ding et al., 2014). However, the restoration of axonal functions in RGCs following treatment with iPSCs was limited in this study. Duplication of TANK-binding kinase 1 (*TBK1*) genes induced by optineurin mutations plays a significant role in glaucoma (Minegishi et al., 2016). In our previous studies, we observed that the TBK1 inhibitor BX795 and *Tbk1* siRNA prevent via p16INK4a inhibition RGC senescence in a mouse model of glaucoma, thus, providing a promising target for a molecular glaucoma therapy (Li et al., 2017).

Several genome-wide association studies found that common variants near the ATP-binding cassette (ABC) transporter A1 (*ABCA1*) gene are associated with glaucoma (Chen Y. et al., 2014; Gharahkhani et al., 2014; Hysi et al., 2014). ABCA1 belongs to a large superfamily of ABC transmembrane transporters that mediate cholesterol efflux to lipid-free apolipoprotein AI and apolipoprotein E (Wang and Smith, 2014). ABCA1 is transcriptionally regulated by the liver X receptor (LXR), a member of the nuclear receptor superfamily, with TO901317 being an LXR agonist. In the ophthalmological research field, Yang et al. reported that an increase of ABCA1 levels with TO901317 reduces ocular inflammation in an experimental model of autoimmune uveitis (Yang et al., 2014). In addition, Zheng et al. found that TO901317 protects against N-Methyl-D-aspartate-induced retinal damage through inhibition of nuclear factor κB and amyloid β formation (Zheng et al., 2015). However, the function of ABCA1 in animal models of glaucoma is still unclear.

Previous studies demonstrated a role for ABCA1 in annexin A1 (ANXA1) externalization (Chapman et al., 2003; Omer et al., 2006). ANXA1 that translocates to the cell membrane interacts with formyl peptide receptors (FPRs) and is involved in inflammation, neuroendocrine system regulation, skeletal muscle differentiation, and cancer progression (Boudhraa et al., 2016). Nuclear translocation of ANXA1 directly regulates the expression of the pro-apoptosis gene *Bid* (Li et al., 2016). Our previous study found that nuclear translocation of ANXA1 is associated with glaucoma-induced apoptosis of RGCs (Zhao et al., 2017). However, in mouse models of glaucoma, it is still unclear whether ABCA1 participates in ANXA1 membrane translocation, and whether and how blocking TBK1 directly protects against RGC apoptosis.

## Materials and methods

### Animals

Male C57BL/6J mice (3 months, 20–25 g; The Experiment Animal Center of the Tongji Medical College, Huazhong University of Science and Technology, China) were housed in covered cages, fed with a standard rodent diet ad libitum, and kept on a 12 h light/12 h dark cycle. All procedures concerning animals were performed in accordance with the Association for Research in Vision and Ophthalmology (ARVO) statement for the Use of Animals in Ophthalmic and Vision Research and under protocols approved by the Institutional Animal Care and Use Committees at Huazhong University of Science and Technology (2015-K-023).

### Reagents and antibodies

The non-selective LXR agonist TO901317 was obtained from Sigma-Aldrich (T2320; China). MG132 was purchased from Calbiochem (474790, WI, USA). The FPR pan-antagonist N-tert-butoxycarbonyl-L-Phe-D-Leu-L-Phe-D-Leu-L-Phe (Boc2) was obtained from MP Biomedicals (02152760; Solon, OH, USA). Terminal deoxynucleotidyl transferase-mediated nick end labeling (TUNEL) assay kit was provided by Roche (11684817910, Basel, Switzerland).

The primary antibodies used in this study are shown in Table 1.

**Table 1:** Antibodies employed in the study.

**Antibody**	**Type**	**WB**	**IH**	**IF**	**IP**	**Source**
Flag (DYKDDDDK tag)	mAb	1:1000			1:50	Cell signaling (#14793)
IL-17	pAb	1:500	1:100			Santa cruz (sc-7927)
IL-1β	pAb	1:500	1:200			Santa cruz (sc-7884)
Iba1	pAb	1:500	1:200			Abcam (ab5076)
β-actin	mAb	1:1000				Santa cruz (sc-47778)
Anti-beta 1 sodium potassium ATPase	mAb	1:250		1:200		Abcam (ab-2873)
Annexin A1	mAb	1:2000		1:1000		Abcam (ab-214486)
ABCA1	mAb	1:500	1:200	1:200		Abcam (ab-18180)
TBK1	mAb			1:100		Abcam (ab-40676)
GFP	mAb	1:3000			1:200	Abcam (ab6556)
Phospho-(Ser/Thr)	pAb	1:1000				Cell signaling (#9631)

*WB, Western blotting; IH, Immunohistochemistry; IF, Immunofluorescence; IP, Immunoprecipitation*.

### Ischemia-reperfusion mouse model

The mice were anesthetized by intraperitoneal injections of 10 mL/kg of 4% chloral hydrate. The corneas were topically anesthetized with 0.5% tetracaine hydrochloride, and the pupils were dilated with 1% tropicamide. A 30-G needle was inserted into the anterior chamber of the right eye that was connected via flexible tubing to a saline reservoir. By raising the reservoir, the IOP was elevated to 75 mmHg and maintained at this value for 45 min. Retinal ischemia was confirmed by whitening of the iris and the loss of the red reflex. The subsequent reperfusion was evident from the return of this reflex. After 45 min, the needle was withdrawn, and tobramycin was applied to avoid bacterial infection. The left eye served as a control. Mice were sacrificed 48 h after this procedure.

### Intravitreal injections

The experimental eyes were injected with TO901317 (30 μM/2 μL), Boc2 (5 mg/kg, 2 μL), vehicle (DMSO, 2 μL), or *Tbk1* siRNA (2 μL) into the vitreous cavity through a 35-G needle with a 10-μL Hamilton microsyringe (Hamilton, Reno, NV, USA) before the onset of reperfusion. Tobramycin was applied to prevent bacterial infection.

### Immunofluorescence

The paraffin-embedded retinal sections (5 μm) were gently washed twice with phosphate-buffered saline (PBS) preheated to 37°C. Afterward, the sections were treated again with 5% bovine serum albumin (BSA) for an additional 60 min to block nonspecific binding, before incubating them with rabbit polyclonal TBK1 antibody, rabbit monoclonal ANXA1 antibody, mouse monoclonal ABCA1 antibody, or mouse monoclonal anti-beta 1 sodium-potassium ATPase in 5% BSA at 4°C overnight (Table 1). Retinal sections were then washed and incubated with a conjugated secondary antibody (1:200; Invitrogen) in 5% BSA at 37°C for 1 h. A confocal microscope (Zeiss 510 Meta, Zeiss, Germany) was used to acquire fluorescence images.

### Tunel assay

Apoptotic cells were visualized using the TUNEL assay of an *in situ* Cell Death Detection Kit according to the manufacturer's instructions. Briefly, paraffin-embedded tissue sections were deparaffinized, rehydrated, and fixed in 4% paraformaldehyde solution. After rinsing with PBS twice, the tissue sections were incubated with 20 μg/mL proteinase K for 10 min and refixed in paraformaldehyde solution for 5 min at 24°C. Afterward, the tissue sections were rinsed with PBS and incubated at 37°C for 60 min in a humidified chamber with the TUNEL reaction mix, in order to allow the end-labeling reactions to occur. The sections were then immersed in saline sodium citrate for 15 min at room temperature to terminate the reaction, which was followed by the immersion in 0.3% hydrogen peroxide in PBS for 5 min at room temperature. In accordance with the manufacturer's instructions, the TUNEL reaction mixture was freshly prepared for each experiment; the tissue sections were incubated with a total volume of 100 μL of the reaction mixture for 1 h at room temperature in the dark. Following this, the sections were rinsed with 0.1 M PBS supplemented with 4′,6-diamidino-2-phenylindole (DAPI) and then observed under a fluorescence microscope. TUNEL-positive cells in the ganglion cell layer were counted in eight viewing fields from retinal sections per condition (*n* = 6 retinas) by two investigators in a blinded manner, and the scores were averaged.

### Cell culture

HEK293 cells and BV2 cells were maintained in Dulbecco's modified Eagle's medium (DMEM) supplemented with 10% fetal bovine serum (Gibco), penicillin (100 U/mL), and streptomycin (100 μg/mL) at 37°C in an incubator equilibrated with 5% CO_2_. Confluent cell layers were split two times per week.

### Immunopanning and primary RGCs cultures

Primary cultured RGCs were purified using a two-step immunopanning method according to a published protocol (Winzeler and Wang, 2013). In short, the retinas were digested with papain (16.5 units/mL) and triturated. The cell suspension was first incubated on a panning plate coated with goat anti-rabbit IgG (Thermo Fisher Scientific, Waltham, MA, USA). The nonadherent cells were incubated on a second panning plate coated with goat anti-mouse IgM and mouse anti-Thy1.1 antibodies. Then, the plate was washed with PBS, and the adherent RGCs were released by treatment with 0.125% trypsin. The isolated RGCs were suspended in the medium as described in the above-mentioned publication. Finally, 96-well culture plates were coated with poly-D-lysine and laminin (Sigma-Aldrich). The RGCs were plated at a density of 5,000 cells/well and cultured for at least 10 days prior to further experimental procedures.

### *In vitro* migration assays

For transwell migration assays, the chambers (24-well inserts, 8 μm pore size; BD Bioscience) were placed in DMEM containing 0.1% BSA and incubated at 37°C for 2 h to hydrate and block the membrane. BV2 cells were washed with DMEM and counted. Purified RGCs with RGC-conditioned medium (RCM) were placed in the lower well. For experiments using TO901317 and Boc2, media in the lower chambers (RGCs) were supplemented with 10 μM TO901317, or/and 10 μM Boc2 incubated with BV2 respectively. After 24 h incubation at 37°C and 5% CO_2_, all cells that did not migrate through the pores were removed by a cotton swab. Cells on the lower surface of the membrane were fixed with 4% paraformaldehyde and then stained with mouse monoclonal ionized calcium-binding adapter molecule 1 (Iba1) antibody. The migrated cells were investigated using bright field microscopy.

### Plasmid construct and *TBK1* siRNA

DNA fragments corresponding to the full-length *TBK1* were amplified by polymerase chain reaction (PCR), followed by cloning into the pEGFP-N1 vector (Invitrogen). Mouse *Tbk1* siRNA plasmids were purchased from GeneChem (Shanghai, China). HEK293 cells were then transfected using Lipofectamine 3000 reagents (Invitrogen).

The siRNA sequence that targeted the *Tbk1* sequence (GenBank No.NM_019786) was designed as follows: *5*′*-GTTTAAAGATAAGTCGGAA*′*-3*.

### Reverse transcriptase PCR (RT-PCR) analysis

Total RNA was isolated from mouse retinas using TRIzol reagent (Invitrogen). Total RNA (1 μg) of each sample was reverse-transcribed using EasyScript First-Strand cDNA Synthesis SuperMix (TransGen) in a 20-μL volume. According to the manufacturer's instructions, the PCR amplification was carried out in a total volume of 25 μL containing 0.5 μL of each primer, 1 μL of cDNA, 12.5 μL Taq Master Mix, and H_2_O to 25 μL. Murine *Gapdh* was amplified as a reference standard. Primers were used as follows: *Gapdh* forward, *5*′*-GACAAAATGGTGAAGGTCGGT-3*′*; Gapdh* reverse*, 5*′*-GAGGTCAATGAAGGGGTCG-3*′*; Abca1* forward, *5*′*-ATTCAGCTTGGTGATGCGvGA-3*′*; Abca1* reverse*; 5*′*-CCAAGCTGTCAAGCAACACT-3*′. The melt curves were analyzed to verify that only a single product per primer was amplified. The gene expression was calculated by the delta-delta Ct method. The results were expressed as fold changes in the target gene normalized to the reference gene *Gapdh*.

### Co-immunoprecipitation (Co-IP)

Briefly, cell lysates were generated by sonication in a buffer containing 20 mM 4-(2-hydroxyethyl)-1-piperazineethanesulfonic acid (HEPES), 400 mM KCl, 5% glycerol, 5 mM ethylenediaminetetraacetic acid (EDTA), 0.4% NP-40, and protease inhibitors, and precleared by centrifugation. The cell lysates were then incubated with an anti-ABCA1 antibody overnight at 4°C. The reaction mixture was afterward incubated with protein A/G PLUS-Agarose beads (sc-2003; Santa Cruz) for 2 h at 4°C. The precipitates were washed three times with wash buffer and then eluted from the protein A/G PLUS-Agarose beads by boiling with 1× sodium dodecyl sulfate (SDS) for 5 min at 95°C. The protein samples were resolved by SDS-polyacrylamide gel electrophoresis.

### Protein extraction and preparation

Protein samples were prepared using the Membrane and Cytosol Protein Extraction Kit (P0033; Beyotime, China) according to the recommended protocol. Briefly, cultured HEK293 cells were washed with ice-cold PBS, harvested using a cell scraper, and centrifuged at 3,000 *g* for 5 min. Cell pellets were then resuspended in a membrane and cytoplasmic extraction reagent containing phosphatase inhibitors Phenylmethanesulfonyl fluoride, and protease inhibitors, and incubated on ice for 30 min. Lysates were then centrifuged at 12,000 *g* at 4°C for 10 min to obtain membrane-bound and cytoplasmic protein fractions for later expression analysis.

### Western blot analysis

For analysis of protein expression, total protein was isolated and harvested from retina samples, cultured HEK293 cells, or primary RGC cells at defined time-points. Samples were separated using 6, 10, or 12% polyacrylamide gels and transferred to polyvinylidene difluoride (PVDF) membranes. PVDF membranes were blocked with 5% BSA at room temperature for 60-90 min and afterward incubated overnight at 4°C with antigen-specific primary antibodies (Table 1). Blots were incubated with species-specific horseradish peroxidase-conjugated secondary antibodies for 60 min at room temperature. Proteins were visualized using a chemiluminescence substrate kit (ECL Plus; PerkinElmer Inc, Covina, CA, USA). Target proteins were quantified using ImageJ (NIH, Bethesda, MD, USA) software. These values were normalized to the expression levels of β-actin or anti-beta 1 sodium-potassium ATPase.

### Statistical analysis

Data are expressed as mean ± standard deviation (S.D.) The statistical analysis was performed with GraphPad Prism software (version 5.0, GraphPad Software Inc.) Data were analyzed by one-way analysis of variance (ANOVA) and Student's *t* test in order to determine statistically significant differences. *P* < 0.05 was considered statistically significant.

## Results

### ABCA1 expression is decreased in a mouse model of ischemia-reperfusion

**Figure 1:**
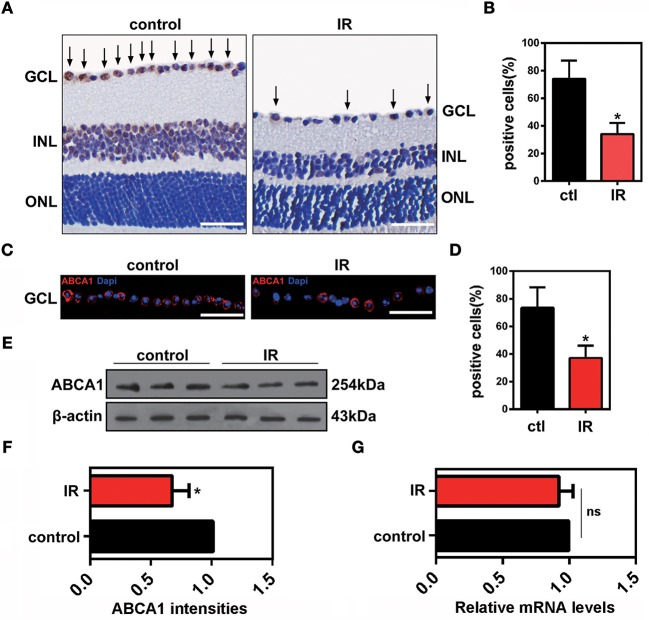
The effects of ischemia-reperfusion on ABCA1 expression *in vivo*. **(A)** Representative immunohistochemistry images of ABCA1 expression in ischemic retina slices. Arrows indicate ABCA1-positive cells. GCL, ganglion cell layer; INL, inner nuclear layer; ONL, outer nuclear layer. Scale bar = 50 μm. **(B)** Statistical analysis of **(A)**. The data are presented as the mean ± S.D. (*n* = 6 retinas per group). *P < 0.05, unpaired Student's *t* test vs. control. **(C)** Immunofluorescence analysis showing the expression of ABCA1 in a retina after IR. A representative result from three independent experiments is shown. Blue: DAPI; red: ABCA1. Scale bar = 50 μm. **(D)** Statistical analysis of **(C)**.The data are expressed as the means ± S.D. (*n* = 6 retinas per group). *P < 0.05, unpaired Student's *t* test vs. control. **(E)** Western blot analysis showing the expression of ABCA1 in a retina after IR. These results were obtained in three independent experiments. **(F)** Statistical analysis of **(E)**. The data are expressed as the means ± S.D. (*n* = 3 retinas per group). *P < 0.05, unpaired Student's *t* test vs. control. **(G)** RT-PCR analysis of *Abca1* gene expression in ischemic retinas 48 h after reperfusion. There was no difference between *Abca1* mRNA levels in the IR and control groups (*n* = 3 retinas per group). *P* > 0.05, unpaired Student's *t* test.

To investigate the change of ABCA1 expression in RGCs after ischemia-reperfusion, we used a mouse model of ischemia induced by acute IOP elevation. This involved an IOP increase to 75 mmHg for 45 min and a reperfusion phase for 48 h. As shown in Figure 1, both immunohistochemical and immunofluorescence analyses revealed that expression of ABCA1 was significantly lower in the IR groups than in the control groups (Figures 1A–D). IR reduced the expression of ABCA1 specifically in the ganglion cell layer GCL (Figure 1A, Supplementary Figure 1B). Western blot analysis of retina extracts also confirmed that compared to the control groups the expression of ABCA1 in the IR groups was significantly decreased (Figures 1E,F). However, there was no difference in mRNA levels between control and IR groups (Figure 1G).

### The LXR agonist TO901317 decreases the cell apoptosis of RGCs after IR

**Figure 2:**
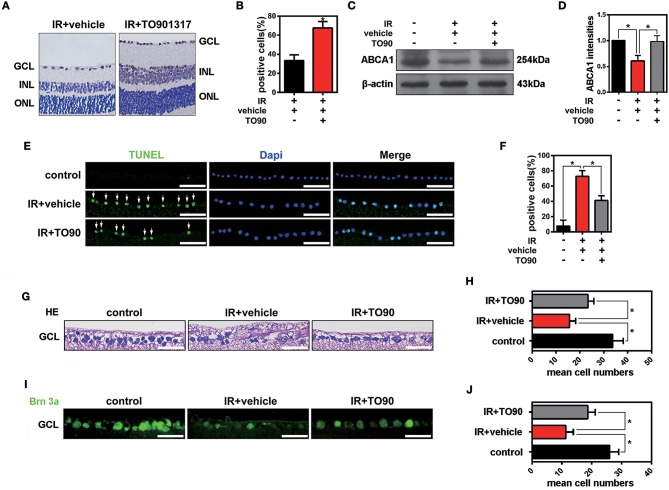
The effects of TO901317 on RGC death after IR *in vivo*. **(A)** Representative immunohistochemical images of ABCA1 expression in the experimental groups IR+vehicle and IR+TO901317 (30 μM). Arrows indicate positive labels. Scale bar = 50 μm. **(B)** Statistical analysis of **(A)**. The data are presented as the mean ± S.D. (*n* = 6 retinas per group). *P < 0.05, unpaired Student's *t* test vs. IR+vehicle. **(C)** Western blot showing the expression of ABCA1 after IR with and without TO901317 pretreatment of the retina. **(D)** Statistical analysis of **(C)**. The data are presented as the mean ± S.D. (*n* = 3 retinas per group). *P < 0.05, one-way ANOVA with Bonferroni *post hoc* test. **(E)** TUNEL staining showing apoptotic cells in the GCL after IR. Please note that TO901317 decreases RGC apoptosis. Arrows indicate positive labels. The statistical analysis is shown in **(F)** (*n* = 6 retinas per group). A one-way ANOVA with Bonferroni *post hoc* test was used. Scale bar = 50 μm. **(G)** Representative HE stainings of retinas show the degeneration of RGCs after IR with and without TO901317 pretreatment. Scale bar = 50 μm. **(H)** Statistical analysis of three independent experiments as shown in **(G)**. The data are expressed as the means ± S.D. (*n* = 6 retinas per group). *P < 0.05, one-way ANOVA with Bonferroni *post hoc* test. **(I)** Representative immunofluorescence images show the Brn-3a staining in the GCL. The statistical analysis of three independent experiments is shown in **(J)** (*n* = 6 retinas per group). *P < 0.05, one-way ANOVA with Bonferroni *post hoc* test. Scale bar = 50 μm.

The LXR agonist TO901317 (30 μM) was injected into the vitreous chambers of mice exposed to IR to induce ABCA1 expression. In accordance with previous experiments, we found that compared with IR+vehicle groups, IR+TO901317 groups effectively increased ABCA1 expression (Figures 2A,B). Western blot results also confirmed that TO901317 could induce the expression of ABCA1 in the retina extract from mice exposed to IR (Figures 2C,D). Cell apoptosis analysis by TUNEL staining revealed that the RGC apoptosis rate was significantly higher in the IR+vehicle group, whereas the LXR agonist decreased this rate after IR induction (Figures 2E,F). Hematoxylin-eosin (H&E) stainings of murine retinas revealed that IR+vehicle groups exhibited a decreased cell number in the GCL, whereas TO901317 reversed this cell loss (Figures 2G,H). Further, we used an antibody against Brn-3a to detect the number of RGCs in the GCL. We found that IR+vehicle groups displayed an RGC loss in the GCL, while TO901317 reverted this RGC loss in the retina slices (Figures 2I,J). These data indicate that TO901317 reduces RGC apoptosis in retinas after IR damage.

### The LXR agonist TO901317 promotes membrane translocation of ANXA1

**Figure 3:**
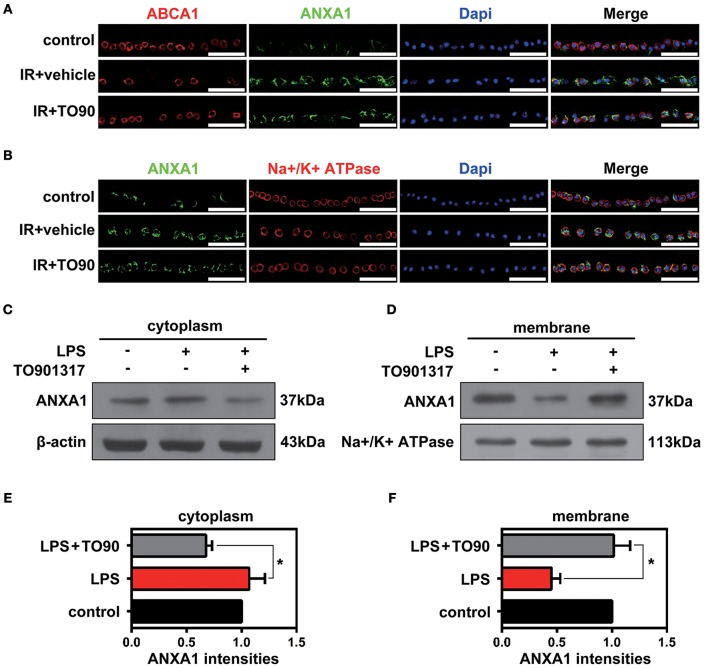
The effects of TO901317 on the ANXA1 translocation to the cell membrane. **(A)** Immunofluorescence results indicate that ABCA1 and ANXA1 can be co-localized on the membrane of cells in the GCL (*n* = 6 retinas per group). Blue: DAPI; red: ABCA1; green: ANXA1. Scale bar = 50 μm. **(B)** TO901317 promotes translocation of ANXA1 to the cell membrane after IR *in vivo* (*n* = 6 retinas per group). Blue: DAPI; red: Na^+^/K^+^-ATPase; green: ANXA1. Scale bar = 50 μm. **(C,D)** Western blots demonstrate that TO901317 (3 μM) increases the protein levels of ANXA1 in the membrane of LPS-stimulated HEK293 cells, while ANXA1 expression in the cytoplasm is reduced. **(E,F)** Statistical analysis of the data shown in **(C,D)**. The data from three independent experiments are expressed as the means ± S.D. *P < 0.05, one-way ANOVA with Bonferroni *post hoc* test.

Previous studies proved that in pituitary folliculostellate cells, the membrane transport of the anti-inflammatory factor ANXA1 is dependent on ABCA1 expression (Chapman et al., 2003; Omer et al., 2006). To observe the effects of ABCA1 on mediating membrane translocation ANXA1 in RGCs, we performed immunofluorescence stainings of retinal slices and extracted cytoplasm and membrane proteins for western blot analyses. Here we used anti-beta 1 sodium-potassium ATPase antibodies to label cell membranes in the GCL. The immunofluorescence analysis indicated that ABCA1 and ANXA1 co-localized on cell membranes in the GCL. However, IR reduced the membrane localization of ANXA1 as well as the co-localization of ABCA1 and ANXA1. Moreover, TO901317 promoted the ABCA1 expression, the ABCA1-ANXA1 co-localization, and the ANXA1 translocation to the cell membrane after IR *in vivo* (Figures 3A,B, Supplementary Figure 1A). Western blotting analyses demonstrated that TO901317 (3 μM) increased the protein levels of ANXA1 in membrane extracts of lipopolysaccharide (LPS)-stimulated HEK293 cells, while ANXA1 expression in the cytoplasm was reduced (Figures 3C–F).

### ABCA1 interacts with ANXA1

**Figure 4:**
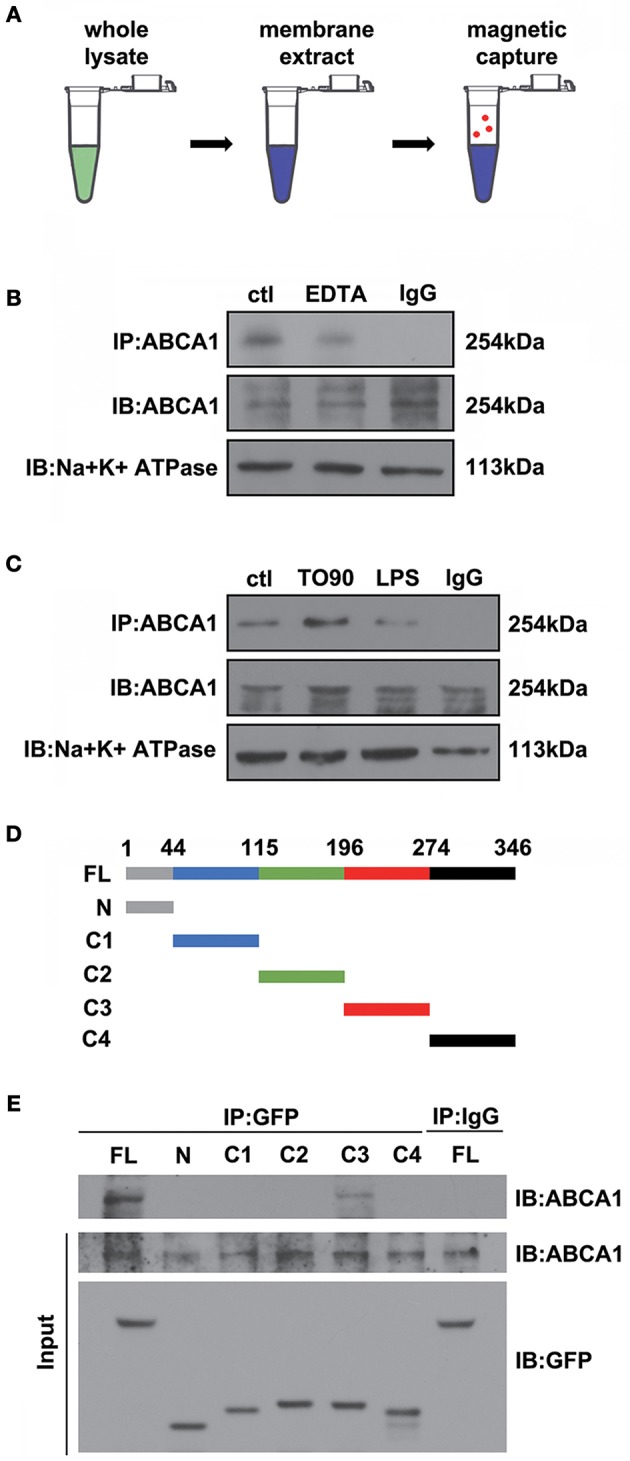
The interaction between ABCA1 and ANXA1. **(A)** Schematic diagram of Co-IP between ABCA1 and ANXA1. **(B)** Western blotting analysis demonstrating that EDTA decreases the interaction between ABCA1 and ANXA1. **(C)** A western blotting analysis indicates that LPS reduces the interaction between ABCA1 and ANXA1, and this effect is inhibited by TO901317. **(D)** Schematic diagram of full-length and fragment ANXA1 plasmids. **(E)** Co-IP results demonstrate that the binding between ANXA1 and ABCA1 involves the C3 domain of ANXA1 (amino acids 196 to 274).

Previous data have shown that ANXA1 membrane localization is promoted by ABCA1 (Chapman et al., 2003; Omer et al., 2006), however, how ABCA1 induces membrane transport of ANXA1 is still unclear. To identify where and how ABCA1 interacts with ANXA1, we extracted membrane proteins from whole cell lysates and then used Co-IP to examine the binding between ABCA1 and ANXA1 (Figure 4A). Results indicated that EDTA decreased the binding between ABCA1 and ANXA1 (Figure 4B). As shown in Figure 4C, LPS stimulation blocked the interaction, whereas TO901317 increased ANXA1 and ABCA1 binding. We then constructed full-length and fragmented ANXA1 plasmids to explore the binding domain (Figure 4D). Results indicated that the C3 domain of ANXA1 (amino acids 196 to 274) may be responsible for the binding between ANXA1 and ABCA1 (Figure 4E). These data indicate that ABCA1 interacts with the C3 domain of ANXA1, that this interaction is calcium-dependent and that LPS inhibits the ABCA1-ANXA1 binding.

### TO901317 decreases microglia activation via regulation of ANXA1 secretion

**Figure 5:**
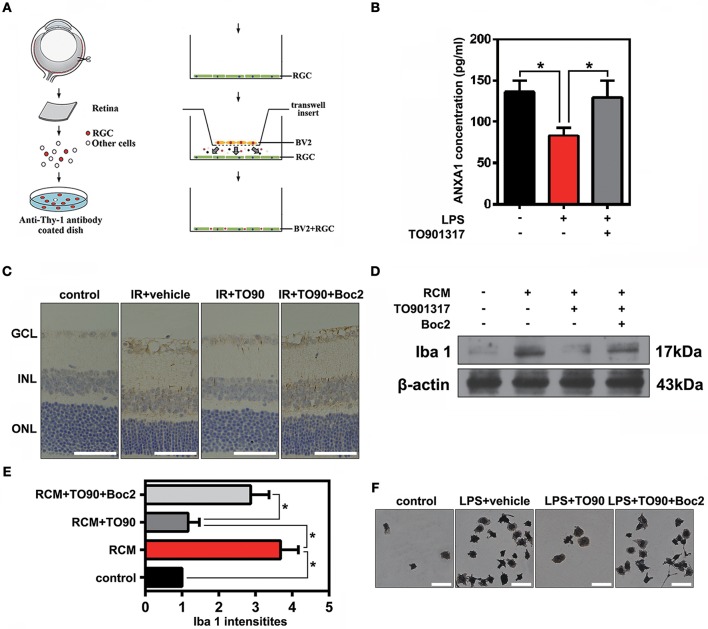
The effects of TO901317 on microglial activation. **(A)** Schematic diagram of immunopanning and transwell migration assays. **(B)** ELISA results reveal that TO901317 leads to an increase of ANXA1 secretion in primary cultured RGCs. A one-way ANOVA with Bonferroni *post hoc* test was used. **(C)** Immunohistochemical results indicate that TO901317 suppresses the activation of microglia after IR *in vivo*, while Boc2 (10 μM) reverses this effect (*n* = 6 retinas per group). Scale bar = 50 μm. **(D)** Western blotting analysis demonstrating that TO901317 reduces the expression of Iba1 in BV2 cells incubated with RCM. This effect is inhibited by Boc2. **(E)** Statistical analysis of the data shown in **(D)**. The data of three independent experiments are expressed as the means ± S.D. *P < 0.05, one-way ANOVA with Bonferroni *post hoc* test. **(F)** TO901317 inhibits microglial migration induced by LPS, and this effect is reversed by Boc2 (10 μM). Scale bar = 20 μm.

To observe the function of ANXA1 in the IR-induced retina damage and microglia activation, co-cultures of primary cultured RGCs and microglia were used in this study. Primary cultured RGCs from murine retinas were isolated by immunopanning using the anti-Thy-1.1 antibody, and then co-cultured with BV2 microglial cells (Figure 5A). Enzyme-linked immunosorbent assay (ELISA) results revealed that LPS stimulation reduced ANXA1 secretion, whereas TO901317 led to an increase of ANXA1 secretion in primary cultured RGCs (Figure 5B). Since Boc2 is a selective blocker of ANXA1 receptors, we investigated whether the administration of Boc2 can block the IR-induced effects of ANXA1 on microglia activation *in vivo* and *in vitro*. Here, we used Iba1 to label activated microglial cells *in vivo* and *in vitro*. Immunohistochemical analyses indicated that TO901317 suppressed the activation of microglia after IR, whereas Boc2 (10 μM) induced their activation (Figure 5C). We collected RCMs from cultures in the presence (RCM+TO901317) and absence of TO901317 and used these media to stimulate BV2 cells. Western blotting analysis demonstrated that TO901317 (3 μM) reduced the expression of Iba1 and that this effect could be inhibited by Boc2 (Figures 5D,E). RGCs and BV2 were incubated with TO901317 and Boc2 for 6 h respectively in the first step, then replaced with normal medium and co-cultured for 24 h, after that BV2 migration was assessed via Iba1 immunohistochemical staining. We found that TO901317 inhibited microglial migration induced by LPS and that this effect could be reversed by Boc2 (Figure 5F). These results indicate that TO901317 downregulates microglial activation via regulation of ANXA1 secretion.

### TO901317 decreases retina inflammation via ANXA1

**Figure 6:**
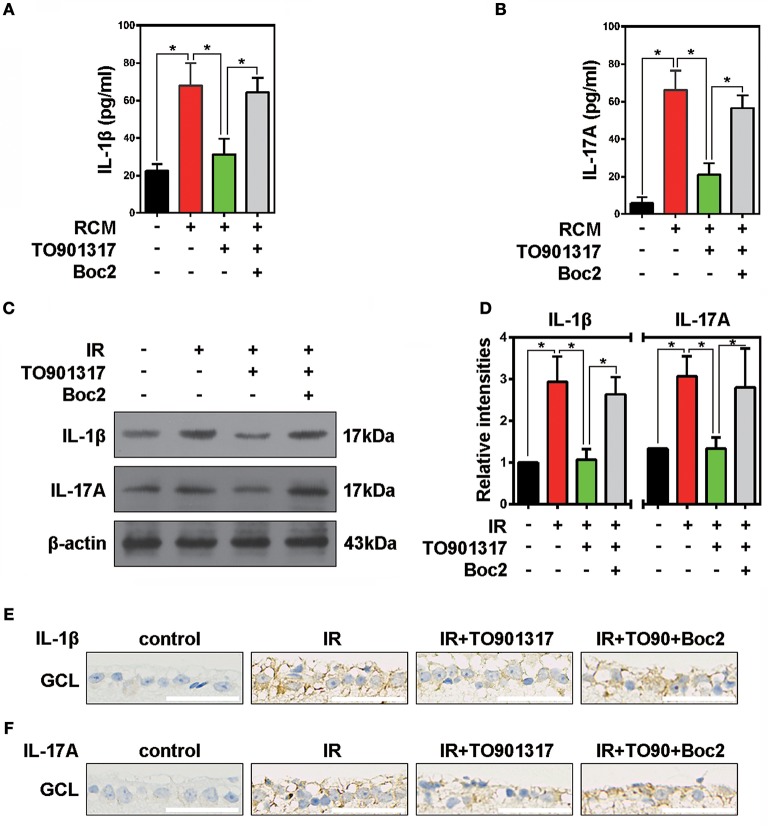
The effects of TO901317 on retinal inflammation. **(A)** ELISA results show the secretion of IL-1β by primary cultured RGCs after RCM or RCM+TO901317 stimulation. The data of three independent experiments are expressed as the means ± S.D. *P < 0.05, one-way ANOVA with Bonferroni *post hoc* test. **(B)** Secretion of IL-17A by primary cultured RGCs detected in ELISAs. The data of three independent experiments are expressed as the means ± S.D. *P < 0.05, one-way ANOVA with Bonferroni *post hoc* test. **(C)** Western blots showing the effects of TO901317 and TO901317+Boc2 on IL-17A and IL-1β expression in ischemic retinas. **(D)** Statistical analysis of the data shown in **(C)**. The data of three independent experiments are expressed as the means ± S.D. *P < 0.05, one-way ANOVA with Bonferroni *post hoc* test. **(E)** Representative immunohistochemical images of IL-1β expression in the GCL. Scale bar = 50 μm. **(F)** Representative immunohistochemical images of IL-17A expression in the GCL. Scale bar = 50 μm.

It is well recognized that microglia activated by external stimuli secrete a large number of pro-inflammatory cytokines (e.g., interleukin [IL]-1β) (Ramirez et al., 2017; Song et al., 2017). Here, we collected RCM and TO901317 incubated RGCs mediums (RCM+TO901317) to stimulate BV2 microglia and used Boc2 to block the function of ANXA1 in BV2. ELISA results revealed that stimulation of BV2 cells with RCM increased their IL-1β and IL-17A secretion, which was reduced by TO901317. However, Boc2 reversed this TO901317 effect in BV2 microglia (Figure 6A,B). Western blot analyses revealed that the elevated IOP increased the expression of IL-17A and IL-1β in the IR group, which could be reduced by TO901317, whereas this TO901317 effect was reversed by Boc2 administration (Figures 6C,D). Similarly, immunohistochemical analyses showed that TO901317 alleviated the IL-1β and IL-17A expression in the GCL, while this effect was reverted by Boc2 (Figures 6E,F). These data indicate that TO901317 reduces via ANXA1 the secretion and expression of the pro-inflammatory cytokines IL-1β and IL-17A in IR-induced retina injury.

### TBK1 stimulates ABCA1 degradation by the ubiquitin-proteasome system

**Figure 7:**
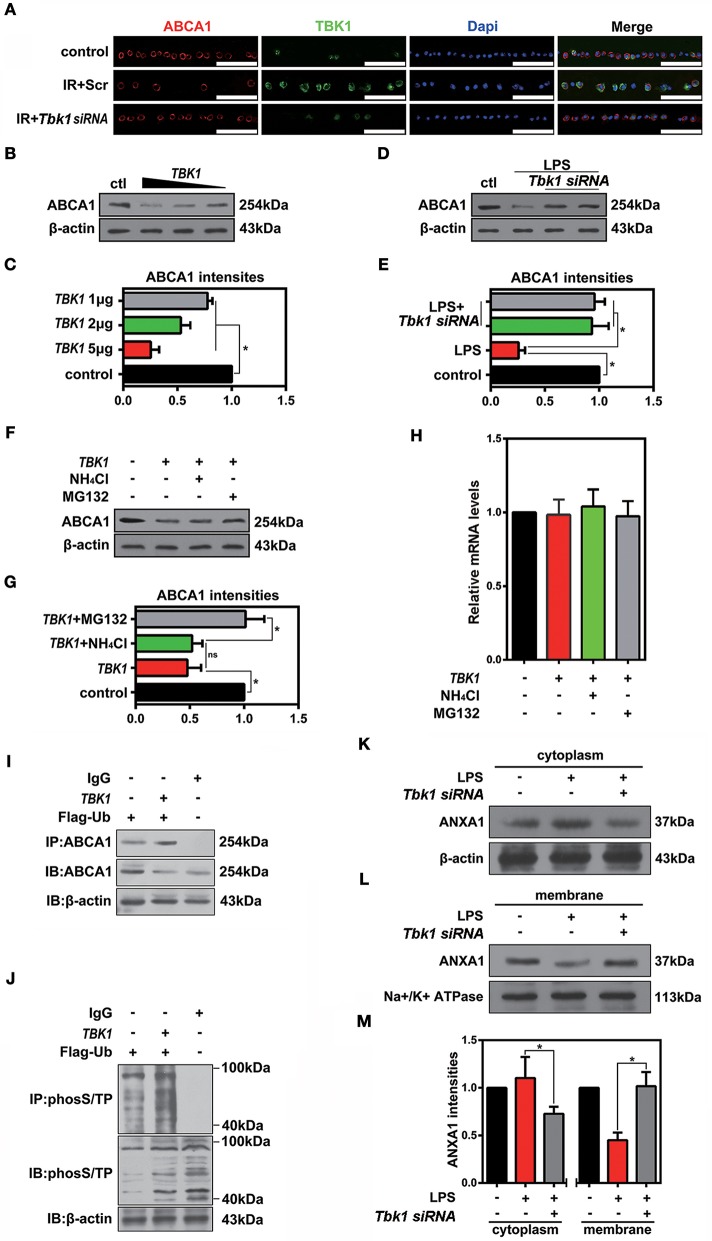
The effects of TBK1 on ABCA1 expression and degradation. **(A)** Immunofluorescence results showing that TBK1 and ABCA1 can be co-localized and that *Tbk1* siRNA knockdown increases the expression of ABCA1 in RGCs (*n* = 6 retinas per group). Blue: DAPI; red: ABCA1; green: TBK1. Scr, Scrambled siRNA. Scale bar = 50 μm. **(B)** Western blots showing the dose-dependent decrease in ABCA1 expression in HEK293 cells after *TBK1* overexpression. **(C)** Statistical analysis of the data shown in **(B)**. The data of three independent experiments are expressed as the means ± S.D. *P < 0.05, one-way ANOVA with Bonferroni *post hoc* test vs. control. **(D)** Western blot showing *Tbk1* siRNA knockdown reverse the reduction of ABCA1 which stimulated by LPS (1 μg/mL). **(E)** Statistical analysis of the data shown in **(D)**. The data of three independent experiments are expressed as the means ± S.D. *P < 0.05, one-way ANOVA with Bonferroni *post hoc* test. **(F)** Western blot showing that Mg132 (10 μM) can prevent in HEK293 a decrease in ABCA1 levels induced by *TBK1* overexpression. **(G)** Statistical analysis of the data shown in **(F)**. The data of three independent experiments are expressed as the means ± S.D. *P < 0.05, one-way ANOVA with Bonferroni *post hoc* test. **(H)** RT-PCR results show the expression of *ABCA1*. The data of three independent experiments are expressed as the means ± S.D. *P* > 0.05, one-way ANOVA with Bonferroni *post hoc* test. **(I)** Co-IP results indicate that ABCA1 ubiquitination is increased following *TBK1* overexpression. **(J)** Representative Co-IP results show that phospho-(Ser/Thr)-linked ubiquitination is increased following transfection with *TBK1*. **(K,L)** Western blots reveal that *Tbk1* siRNA increases the ANXA1 expression in the cell membrane after LPS stimulation, while ANXA1 expression in the cytoplasm is decreased. **(M)** Statistical analysis of the data shown in **(K,L)**. The data of three independent experiments are expressed as the means ± S.D. *P < 0.05, one-way ANOVA with Bonferroni *post hoc* test.

Cellular protein homeostasis is maintained by two major degradation pathways, namely the ubiquitin-proteasome system and autophagy. TBK1 has been shown to have a major role in autophagy and mitophagy, chiefly by phosphorylation of autophagy adaptors. However, the function of TBK1 in the ubiquitin-proteasome pathway and in ABCA1 degradation is unclear. To determine the potential relationship between ABCA1 and TBK1, we injected *Tbk1* siRNA (Li et al., 2017) into murine vitreous chambers 10 days before IR and then measured the co-localization of TBK1 with ABCA1 in retinal slices. The results of the immunofluorescence analyses indicated that TBK1 and ABCA1 co-localized and, importantly, that *Tbk1* siRNA increased the expression of ABCA1 in GCL (Figure 7A). As shown in Figures 7B,C, western blot analyses revealed that the expression of ABCA1 was dose-dependently decreased in HEK293 cells after full-length *TBK1* transfection. Western blot analyses also identified that *Tbk1* siRNA reversed the downregulation of ABCA1 after LPS stimulation (1 μg/mL) to imitate cell injury during IR (Figures 7D,E). Western blot results revealed that MG132 (10 μM) could inhibit the decrease in ABCA1 expression induced by *TBK1* overexpression (Figures 7F,G). However, RT-PCR results showed that there is no significant difference between control and *TBK1* overexpression groups in *ABCA1* expression on the mRNA level (Figure 7H). Figures 7I,J revealed that phospho-(Ser/Thr)-linked ubiquitination and binding between ABCA1 and ubiquitin proteasomes were increased following *TBK1* transfection. Finally, we examined the effects of *Tbk1* siRNA on ANXA1 membrane translocation. As shown in Figures 7K–M, *Tbk1* siRNA increased ANXA1 expression in the membranes of LPS-stimulated HEK293 cells, while ANXA1 expression in the cytoplasm was decreased. Together, these results suggest a pivotal role for TBK1 in ABCA1 degradation and function.

## Discussion

Acute glaucoma is a major cause of irreversible blindness and a sight-threatening disease that is characterized by acute retinal ischemic inflammatory injury and the death of RGCs secondary to the elevated IOP. Several studies have reported that ABCA1 participates in the externalization of ANXA1 (Chapman et al., 2003; Omer et al., 2006) and that TO901317 administration protects the retina and preserves visual functions by suppressing inflammation (Yang et al., 2014). In the present study, we found that the expression of ABCA1 was decreased in a mouse model of IR with no difference in its mRNA levels between the control and IR groups. TO901317 increased the expression of ABCA1 and reversed RGC apoptosis and retinal inflammation *in vivo*. ABCA1 interacted with ANXA1 and induced ANXA1 membrane secretion, which determined the activation of retinal microglia. The IR-induced upregulation of TBK1 might be involved in ABCA1 ubiquitination and degradation.

Previous studies identified that ABCA1 plays a major role in high-density lipoprotein formation (Feingold and Grunfeld, 2000; Rosenson et al., 2016). As high-density lipoproteins were considered to have anti-inflammatory and other beneficial effects (Murphy et al., 2012; Mao et al., 2017), ABCA1 was implicated in macrophage-associated inflammatory diseases. Microglia are specialized resident macrophages in the neural retina, and they are normally located in the nerve fiber layer, ganglion cell layer, inner plexiform layer, and outer plexiform layer (Chinnery et al., 2017). In this study, we found that TO901317 increases the ABCA1 expression and, thus, prevents the expression of the pro-inflammatory cytokines IL-1β and IL-17A as well as the apoptosis of RGCs after ischemia-reperfusion injury.

We also investigated the mechanisms of ABCA1 in retinal inflammation. Previous studies have shown that ANXA1 acts via FPRs as an anti-inflammatory factor in the host defense system and the nervous system (Parente and Solito, 2004; Chen L. et al., 2014). Liu et al. found that *Anxa1* knock out increases the expression of the pro-inflammatory cytokines IL-1β, IL-6, and tumor necrosis factor (TNF)-α (Liu et al., 2015). In addition, Luo et al. identified that the FPR antagonist Boc2 downregulates microglia activation (Luo et al., 2014). ABCA1 was known to bind to ANXA1 (Chapman et al., 2003; Omer et al., 2006), but the function and mechanism were not clear. We speculated that ABCA1 might assist in ANXA1 secretion. Co-IP results from membrane extracts revealed that ABCA1 interacts with ANXA1 and that TO901317 increased ABCA1-ANXA1 membrane binding as well as ANXA1 secretion. In addition, we found that pretreatment of primary cultured RGCs with TO901317 decreases the migration of BV2 microglia and reduces their activation and expression of pro-inflammatory factors. All of these effects are reversed by Boc2. These results suggest that ABCA1 plays a role in ANXA1 membrane secretion and in the alleviation of inflammation.

Transcriptional programs operate to mediate the long-term control of ABCA1 (Koldamova et al., 2014). The liver X receptor agonist TO901317 was considered an effective up-regulator of ABCA1 expression. However, the ABCA1 post-translational modifications were unclear, especially as ABCA1 is ubiquitylated and degraded by as yet unknown E3 ligases (Sharpe et al., 2014). Hsieh et al. found that the degradation of ABCA1 is completely inhibited by the proteasome inhibitor MG132 (Hsieh et al., 2014). In our study, we found that the expression of ABCA1 was negatively correlated with TBK1. *TBK1* overexpression increased ABCA1-ubiquitin proteasome binding and ubiquitin-proteasome phosphorylation. The proteasome inhibitor MG132 prevented the ABCA1 degradation induced by *TBK1* overexpression. These results suggest that *TBK1*-induced ABCA1 degradation might occur through the promotion of ubiquitin-proteasome phosphorylation.

The determination of the cell fate by ANXA1 was dependent on the subcellular protein localization not only in cancer cells but also in neural cells. Secreted ANXA1 interacts with FPRs and is involved in inflammation, neuroendocrine system regulation, skeletal muscle differentiation and cancer progression (Boudhraa et al., 2016), while nuclear translocation of ANXA1 is implicated in neuronal apoptosis after stroke (Zhao et al., 2015; Li et al., 2016). Omer et al. demonstrated that ABCA1 is co-localized with ANXA1 on the cell membrane of folliculostellate cells and that ABCA1 is involved in ANXA1 transport across the plasma membrane. In our study, ABCA1 and ANXA1 could be co-localized on the cell membranes of RGCs and, more importantly, administration of both *Tbk1* siRNA and TO901317 increased the expression of ANXA1 on the membranes and decreased the expression in the cytoplasm. More significantly, the Co-IP results indicate that the interactions between ABCA1 and ANXA1 occur on the ANXA1 C3 fragment.

**Figure 8:**
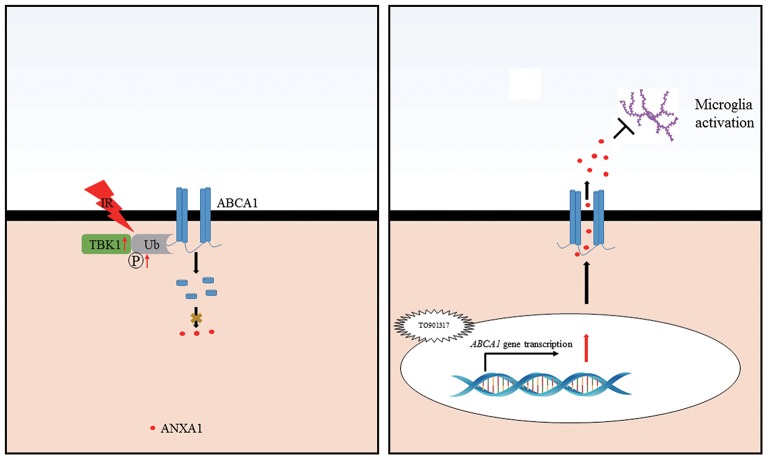
Schematic representation of the cellular events influencing RGC apoptosis in ischemic injury. Ischemia-reperfusion injury induces an increase in TBK1 expression, which promotes ABCA1 ubiquitination and degradation, thus decreasing ANXA1 membrane transport (**Left**). TO901317 administration during ischemia-reperfusion increases the expression of ABCA1. This maintains ANXA1 membrane translocation and secretion, which inhibits microglia activation (**Right**).

Our findings identify a novel mechanism for ABCA1 in glaucoma. Ischemia reperfusion injury decreases ABCA1 expression in the GCL, which causes a decreased ANXA1 translocation to the cell membrane and its secretion. The TBK1 upregulation induced by IR stimulates ABCA1 degradation. TO901317 induces ABCA1 expression, thus, stimulating ANXA1 membrane translocation and secretion, which then prevents microglia activation (Figure 8). These findings provide with ABCA1 and TBK1 novel targets for glaucoma therapies.

## Author contributions

YZ and HZ conceived and designed the study. LL, XL, LX, WC, and YZ performed the experiments. QD, QX, LZ, and HZ assisted with analyzing the data. LL and YZ drafted the manuscript, which was reviewed by and YZ.

### Conflict of interest statement

The authors declare that the research was conducted in the absence of any commercial or financial relationships that could be construed as a potential conflict of interest. The reviewer LP declared a shared affiliation, with no collaboration, with the authors to the handling Editor.

## Supplementary material

Supplementary Figure 1 The expression of ABCA1/ANXA1 in full retinal slice after IR. **(A)** Representative immunofluorescence images of ANXA1 expression in ischemic retina slices. GCL: ganglion cell layer; INL: inner nuclear layer; ONL: outer nuclear layer. **(B)** Immunofluorescence analysis showing the expression of ABCA1 in a full retina slice after IR.
