# Supplementary figures and images for: Reduced Annexin A1 Secretion by ABCA1 Causes Retinal Inflammation and Ganglion Cell Apoptosis in a Murine Glaucoma Model
Source: Front Cell Neurosci. 2018 Oct 11;12:347. doi: 10.3389/fncel.2018.00347 (PMC6193130; doi:10.3389/fncel.2018.00347)

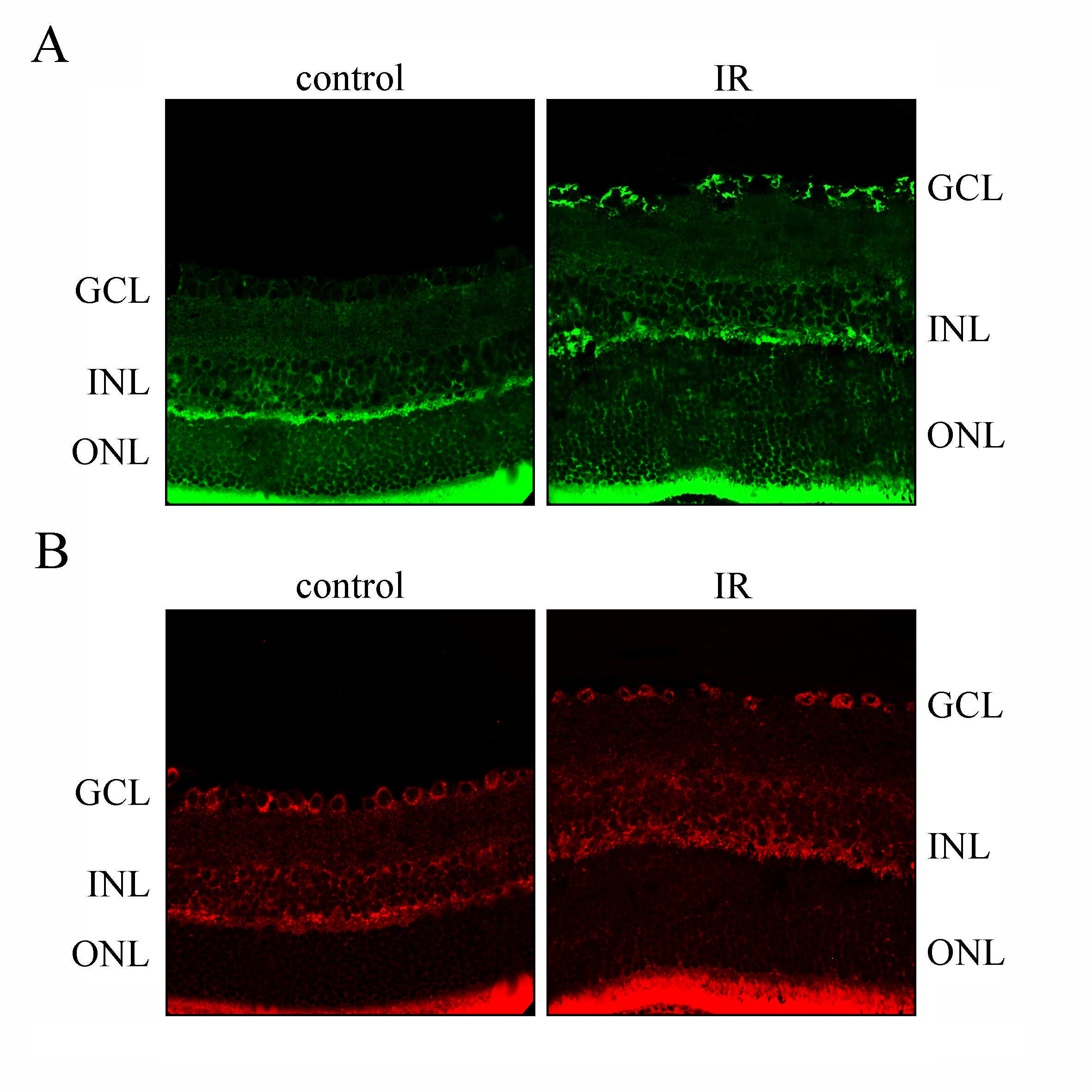

Supplement: Supplementary Figure 1 — The expression of ABCA1/ANXA1 in full retinal slice after IR. (A) Representative immunofluorescence images of ANXA1 expression in ischemic retina slices. GCL: ganglion cell layer; INL: inner nuclear layer; ONL: outer nuclear layer. (B) Immunofluorescence analysis showing the expression of ABCA1 in a full retina slice after IR. [file Image_1.TIF]
